# Impaired Sympathoadrenal Axis Function Contributes to Enhanced Insulin Secretion in Prediabetic Obese Rats

**DOI:** 10.1155/2011/947917

**Published:** 2011-08-16

**Authors:** Ana Eliza Andreazzi, Sabrina Grassiolli, Paula Beatriz Marangon, Adriana Gallego Martins, Júlio Cézar de Oliveira, Rosana Torrezan, Clarice Gravena, Raúl Marcel González Garcia, Paulo Cezar de Freitas Mathias

**Affiliations:** ^1^Laboratory of Physiology, Department of Physiology, Federal University of Juiz de Fora, 36036-900 Juiz de Fora, MG, Brazil; ^2^Laboratory of Secretion Cell Biology, Department of Cell Biology and Genetics, State University of Maringá, 87020-900 Maringá, PR, Brazil; ^3^Laboratory of Cell Biology, Department of Biology, Federal University of Juiz de Fora, 36036-900 Juiz de Fora, MG, Brazil

## Abstract

The involvement of sympathoadrenal axis activity in obesity onset was investigated using the experimental model of treating neonatal rats with monosodium L-glutamate. To access general sympathetic nervous system activity, we recorded the firing rates of sympathetic superior cervical ganglion nerves in animals. Catecholamine content and secretion from isolated adrenal medulla were measured. Intravenous glucose tolerance test was performed, and isolated pancreatic islets were stimulated with glucose and adrenergic agonists. The nerve firing rate of obese rats was decreased compared to the rate for lean rats. Basal catecholamine secretion decreased whereas catecholamine secretion induced by carbachol, elevated extracellular potassium, and caffeine in the isolated adrenal medulla were all increased in obese rats compared to control. Both glucose intolerance and hyperinsulinaemia were observed in obese rats. Adrenaline strongly inhibited glucose-induced insulin secretion in obese animals. These findings suggest that low sympathoadrenal activity contributes to impaired glycaemic control in prediabetic obese rats.

## 1. Introduction

Obesity is a worldwide epidemic and the most important factor in metabolic syndrome onset. This syndrome is part of a group of comorbid pathologies that include insulin resistance, type 2 diabetes, and hypertension [1, 2]. According to the literature, the incidence of these diseases could double by the year 2030 [3]. 

Over the past decade, the central nervous system (CNS) has been recognised as a key player in controlling energy homeostasis [4]. Although other brain areas are important, the hypothalamus has been identified as the pivotal structure regulating food intake and energy balance [5]. The arcuate hypothalamic nucleus (ARC) senses peripheral nutrient and hormonal signals, including insulin and leptin, which are well-known adiposity signals [6, 7]. The ARC integrates these peripheral signals, and this information is relayed to several second-order hypothalamic targets that modulate the autonomic nervous system (ANS) [8, 9].

The ANS plays a key role in homeostatic control, including regulating heart rate, body temperature, blood pressure, and respiration [10]. The ANS is primarily an efferent system that transmits impulses from the hypothalamus to regulate peripheral organ systems. The ANS is further subdivided into two principal components, the parasympathetic (PNS) and sympathetic (SNS) nervous systems. In general, activation of the splanchnic nerve in the SNS mediates catabolic processes, whereas stimulation of the vagus nerve in the PNS mediates anabolic responses [11]. According to Teff, the central control of insulin secretion is a well-known example of these opposing processes, as vagal activation enhances insulin release from pancreatic beta cells, whereas noradrenaline released from the SNS inhibits this process [11]. 

Several studies have demonstrated a relationship between the SNS and obesity [10, 12, 13]. Low SNS activity has been proposed as a risk factor for future body weight gain, as classically demonstrated by reports from Bray and collaborators that were based on experimental evidence in rodents showing that lesions in the hypothalamus lead to low SNS activity and morbid obesity [14, 15]. 

The sympathoadrenal system includes the SNS and the chromaffin cells from the adrenal medulla, which secretes catecholamines (primarily adrenaline) into the bloodstream [16]. Chromaffin cells are cholinergically innervated by the splanchnic nerve from the SNS; acetylcholine released upon stimulation of this nerve activates neuronal cholinergic receptors in chromaffin cells, thereby inducing membrane depolarisation and triggering catecholamine secretion [17]. 

Once secreted, the catecholamines bind to cell-surface *α*- and *β*-adrenergic receptors. In general, activation of *β*-adrenergic receptors induces lipolysis, bronchodilation, vasodilation, thermogenesis, and increased cardiac output; on the other hand, activating *α*-adrenergic receptors induces vasoconstriction and inhibits insulin secretion [18]. High insulin concentrations in fasting and fed states trigger insulin resistance. Increased insulin secretion leads to altered receptor function or postreceptor defects in insulin signalling [19]. Therefore, pharmacotherapeutic approaches that target insulin secretion and/or augment sympathetic output have been pursued in an attempt to either promote weight loss or attenuate weight gain [20]. The purpose of the present study was to investigate sympathoadrenal function and its effect in regulating insulin secretion in pre-diabetic obese rats.

## 2. Material and Methods

### 2.1. Animals

All animal protocols were performed in accordance with the precepts of the Brazilian College of Animal Experimentation (COBEA) and Brazilian Federal Law. Once a day during the first 5 days after birth, monosodium L-glutamate (MSG, 4 mg/g body weight) was injected subcutaneously into the cervical area of Wistar rat pups. Control animals received saline solution. The pups were weaned at 21 days of age, and only males were used in the study. Throughout the protocol period, the animals received water and commercial chow (Nuvital, Curitiba, Brazil) *ad libitum* (except when animals were fasted) and were placed in an environmentally controlled room maintained at 23 ± 3°C with a 12-hour light/12-hour dark photocycle (with the lights on from 07:00 to 19:00 h.).

### 2.2. Obesity

To evaluate the onset of obesity, 90-day-old rats were anaesthetised by a lethal intraperitoneal injection of sodium pentobarbital (45 mg/100 g body weight). The epididymal and retroperitoneal fat pads were removed, rinsed, and weighed to estimate obesity induced by MSG treatment [21]. The Lee index was calculated using the formula [(body weight)^1/3^/(nasoanal length)] × 1000 (where body weight and length are in g and cm, resp.) and used as a predictor of obesity in MSG rodents [22].

### 2.3. Electrical Sympathetic Activity

After fasting for 12 hrs, 8 rats from each experimental group were anesthetised with thiopental (45 mg/kg). Following the method of Leon-Quinto et al., the sympathetic branch nerve from the superior ganglion was dissected and placed on a pair of hook-shaped silver recording electrodes (0.6 mm diameter) [23]. The nerve was covered with silicone oil to prevent dehydration. The electrode was connected to an electronic device (Bio-Amplificator, Insight, Riberão Preto, Brazil) that amplified the electrical signal up to 10,000 times after filtering out low and high frequencies with a 1–80 kHz band-pass filter. The neural output signal was acquired with an Insight interface (Insight), viewed online, and stored on a personal computer using a software program from Insight. During data acquisition, the animals were placed in a Faraday cage to block external electromagnetic interference. After stabilisation, 40 recording frames were analysed from the signal (over 5–10 minutes) for spike counting. Nerve activity was quantified as the number of spikes counted during 5 seconds. For each rat, the average number of spikes per 5 seconds counting trial was used to calculate nerve firing rate.

### 2.4. Adrenal Glands

Both adrenal glands were removed and weighed. During handling, the glands were kept on an ice bath in standard Krebs-HEPES solution containing (in mM) Cl^−^, 154.2; Na^+^, 144.0; Ca^2+^, 2.5; Mg^2+^, 1.18; SO_4_^2−^, 1.2; K^+^, 3.5; glucose, 11.1; HEPES, 25.0; bovine serum albumin (BSA) 0.5%. The right glands from both experimental groups (*n* = 15 for each group) were used to measure the total catecholamine (adrenaline and noradrenaline) content using the trihydroxyindole fluorescence method [24]. The parameters used were 420 nm for excitation and 510 nm for emission. For total catecholamine content, the glands were homogenised in 350 *μ*L of 10% acetic acid using an ultrasonic processor and centrifuged at 10,000 ×g for 1 min. The data were obtained by plotting the values on a linear regression line generated from a standard adrenaline curve. The left adrenal glands were used for experiments measuring secretion (*n* = 10 for each group). The adrenal medulla was dissected using a stereoscopic lens and ophthalmological surgical instruments. To aid in manipulation, the isolated medullae were impaled on steel needles and left to rest for 40 minutes in standard Krebs-HEPES solution.

### 2.5. Stimulation of Catecholamine Secretion

Costar 96-well cell culture plates were used. Each well contained 200 *μ*L of standard Krebs-HEPES or modified Krebs-HEPES solution containing 100 *μ*M carbachol, a synthetic analogue of acetylcholine, 25 mM caffeine, a well-known inducer of calcium release from the endoplasmic reticulum, and elevated (50 mM) K^+^, which triggers plasma membrane depolarisation. The pH of these solutions was maintained between 7.0 and 7.2 at room temperature. The medullae were incubated three times for 5 minutes each in a three-well sequence in standard Krebs-HEPES solution and then incubated for 5 minutes in modified Krebs-HEPES solution. The medullae were then placed in 220 *μ*L of 10% acetic acid and homogenised by sonication (at 60 MHz for 10 min) to extract nonsecreted catecholamines. Acetic acid (20 *μ*L) was added to each well to preserve the secreted catecholamines. Samples of secreted and remaining catecholamines were assayed by the fluorometric method described above.

### 2.6. Western Blot Analysis

Another batch of left adrenal glands (*n* = 10 for each group) was processed for Western blot analysis to quantify tyrosine hydroxylase (TH) and *α*-PKC protein levels as previously reported [25], with some modifications. Briefly, the glands were homogenised in 0.3 mL phosphate buffer (pH 7.4) containing 1 *μ*L protease inhibitor cocktail (1 mg/mL each of aprotinin, leupeptin, and trypsin inhibitor) and centrifuged at 1120 ×g for 15 min at 4°C. Protein concentration in the supernatant was determined by the Bradford method [26]. The proteins in the supernatant were separated by SDS-PAGE using 20 and 100 *μ*g total protein for TH and *α*-PKC, respectively. The proteins were electroblotted to a nitrocellulose membrane (Hybond-P ECL membrane, Amersham Biosciences, UK), and the membranes were incubated with Tris-buffered saline (TBS) containing 5% (w/v) nonfat dry milk for 90 min to block nonspecific binding sites. The membranes were then washed with TBS and incubated overnight at 4°C in primary antibody (monoclonal mouse anti-TH, Sigma-Aldrich, USA, or mouse anti-*α*-PKC, Santa Cruz Biotechnology, USA) diluted at 1 : 2000 in TBS containing 0.5% nonfat dry milk. The membranes were then washed and incubated in secondary HRP-conjugated goat antimouse antibody (Santa Cruz Biotechnology, USA) diluted at 1 : 2000 in TBS containing 0.5% nonfat dry milk for 1 hr at room temperature. Finally, the TH and *α*-PKC bands were visualised by chemiluminescence (ECL Plus Kit, Amersham Biosciences, Sweden) followed by exposure to autoradiographic film (Hyperfilm ECL, Amersham Biosciences). The area and intensity of the bands were quantified using Abeletro software (UFJF-Juiz de Fora, Brazil). Except where stated otherwise, all reagents were purchased from Sigma Chemical Co. (St. Louis, Mo, USA).

### 2.7. Plasma Adrenaline Measurement

Blood samples were collected from 10 rats per group. After separating the plasma (200–300 *μ*L per animal), it was stored under refrigeration and transferred to microtubes containing sodium metabisulfite antioxidant. High-performance liquid chromatography (HPLC) was used to measure the adrenaline contained in 100 *μ*L samples [27].

### 2.8. Glucose Tolerance Test

The intravenous glucose tolerance test (ivGTT) was performed after a 12-hour fast (from 19:00 to 07:00 h). The rats (*n* = 10 for each group) underwent surgery to implant a cannula into the right jugular vein. At 24 hours after surgery, a glucose load (1 g/kg body weight) was delivered throughout the cannula. Animals in a second group received an intraperitoneal injection of the *α*-adrenergic agonist oxymetazoline (Oxy, 16 nM/kg body weight) 5 min prior to the glucose load. Blood samples (300 *μ*L) were collected from the same cannula before the glucose load (t0) and 5 (t5), 15 (t15), 30 (t30), and 60 (t60) min after the glucose injection; the t0 sample was used to measure plasma glucose and insulin concentrations by the glucose-oxidase technique (Kit Bio Diagnostic Chemistry Industry, Paraná-Brazil) and RIA, respectively, [28, 29].

### 2.9. Pancreatic Islet Isolation and Insulin Secretion

Rat pancreatic islets were isolated as previously described [30]. Batches of 4 islets (*n* = 20 batches of islets from 5 different rats) were preincubated for 60 min in 1 mL normal Krebs solution containing (in mM): 120 mM NaCl; 4.8 mM KCl; 2.5 mM CaCl_2_; 1.2 mM MgCl_2_; 24 mM NaHCO_3_; 5.6 mM glucose. This solution was gassed with O_2_/CO_2_ (95/5%) to maintain pH at 7.4 and supplemented with BSA (0.12%, w/v) and used in the following steps. After equilibrating to a low glucose concentration solution, the islets were incubated for 60 min in Krebs solution containing 5.6 or 8.3 or 16.7 mM glucose. After preincubation, other islet batches were incubated for 60 min in the presence of the *α*-adrenergic agonist. Adrenaline (1.0 *μ*M) was added to the islets incubated in the Krebs solution with 16.7 mM glucose and 0.1 *μ*M of the adrenergic *β*-blocker propranolol, which was used to avoid *β*-adrenoceptor potentiation effects on insulin secretion [31]. This concentration of adrenaline (1.0 *μ*M) blocks glucose-induced insulin secretion [32]. Insulin concentrations were measured by RIA from aliquots prepared from the incubations.

### 2.10. Statistical Analysis

All results are presented as the mean ± SEM. Differences were considered statistically significant when *P* < 0.05. One-way ANOVA with Bonferroni post hoc test and Student's *t-*test were performed using GraphPad Prism version 5.00 for Windows (GraphPad Software, San Diego, Calif, USA).

## 3. Results

As shown in Table 1, MSG treatment increased the epididymal and retroperitoneal fat pad mass by 60.5 and 57.2%, respectively, compared with control animals (*P* < 0.001). Indeed, the Lee index increased by 6.9% in obese rats compared with controls (*P* < 0.05). These results confirm the effectiveness of neonatal MSG treatment in inducing adult obesity. Obese rats showed a nearly 3-fold increase in insulinaemia compared with control rats (*P* < 0.05); in contrast, glycaemia was unchanged in both groups. Moreover, adrenal gland mass was reduced 49.5% in pre-diabetic obese rats compared with control rats (*P* < 0.001).

**Figure 1:**
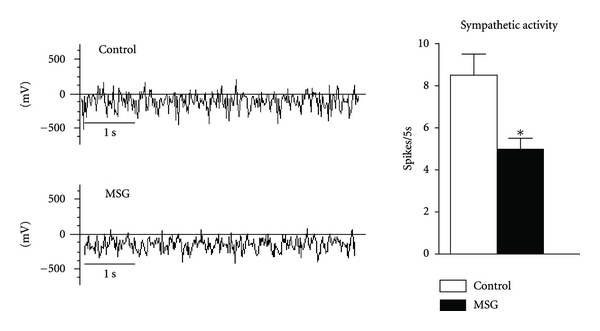
Sympathetic activity in MSG-obese rats. The bars represent the frequency of nerve discharges in mean ± SEM (*n* = 8 animals per group). Student's *t*-test was used. *P < 0.05 compared to control, by Student's *t*-test. Representative records of nerve discharges from a control and MSG-obese animal are shown.

Recordings of sympathetic nerve activity are shown in Figure 1. In both resting, and fasting states, the sympathetic firing rate was 58.8% lower in pre-diabetic obese rats compared with control rats (*P* < 0.05).

**Figure 2:**
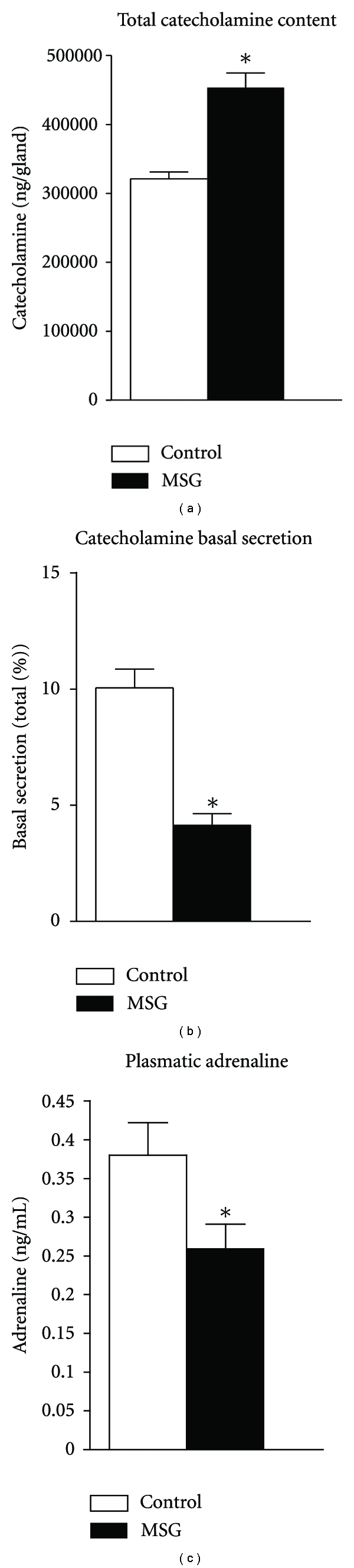
Adrenal medulla total catecholamine content, basal catecholamine secretion, and blood circulating adrenaline concentration in MSG-obese rats. The bars represent mean ± SEM (*n* = 10–15 animals per group). *P < 0.05 compared to control, by Student's *t*-test.

Figure 2 shows total catecholamine stores in the adrenal glands, basal catecholamine secretion from isolated adrenal medullae and fasting plasma adrenaline levels. Total adrenal catecholamine content was increased by 40.3% in pre-diabetic obese rats compared with controls (*P* < 0.01). However, the pre-diabetic rats showed a 60% reduction in basal catecholamine secretion from the adrenal medulla and 32% lower plasma adrenaline concentration compared with the control animals (*P* < 0.05).

**Figure 3:**
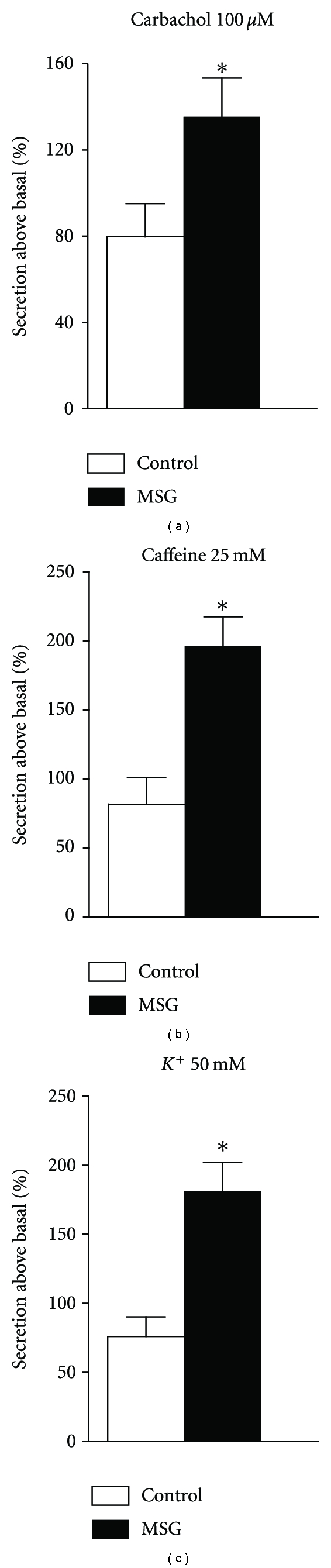
Adrenal catecholamine secretion in MSG-obese rats. The bars represent mean ± SEM (*n* = 8–10 animals per group). *P < 0.05 compared to control, by Student's *t*-test.

Figure 3 shows that carbachol, caffeine, and potassium each induced higher levels of catecholamine release in obese rats compared with control rats. Specifically, carbachol, caffeine, and potassium led to a 1.7-, 2.4-, and 2.5-fold increase in catecholamine secretion, respectively, from pre-diabetic obese adrenal medullae relative to controls.

**Figure 4:**
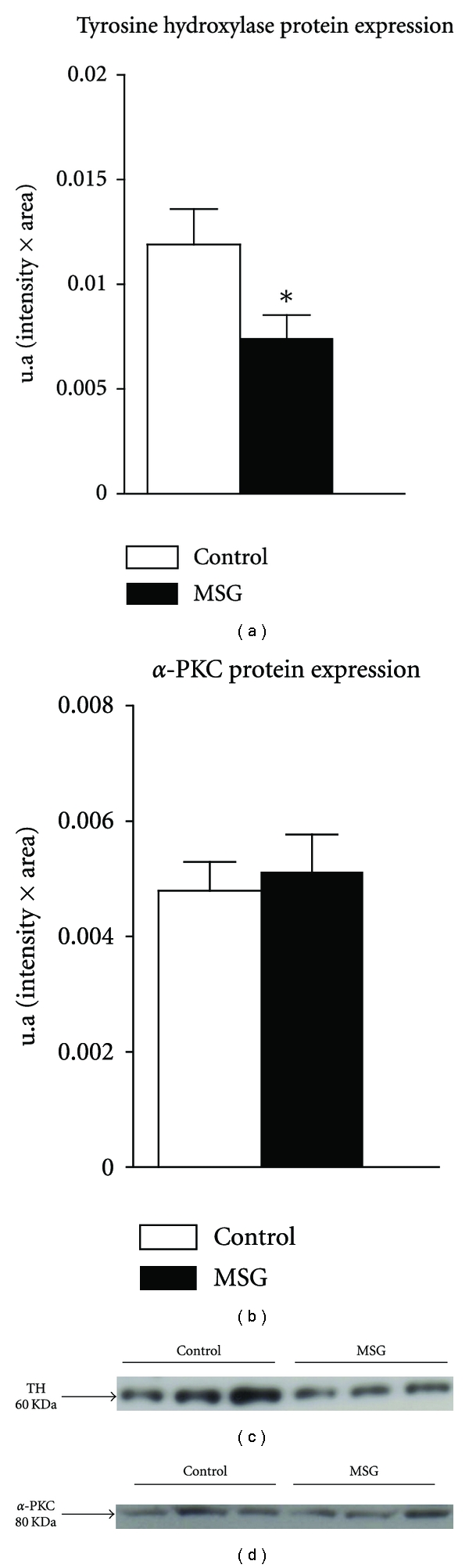
TH and *α*-PKC expression in MSG-obese rats. The (a,b) bars represent mean ± SEM (*n* = 6–9 animals per group). *P < 0.05 compared to control, by Student's *t*-test. Representative Western blots of TH and PKC are shown in the middle and lower panels, respectively.

As shown in Figure 4, TH protein levels decreased 37.6% in pre-diabetic rats compared with control rats (*P* < 0.05). In contrast, *α*-PKC protein levels did not differ between the two groups. 

**Figure 5:**
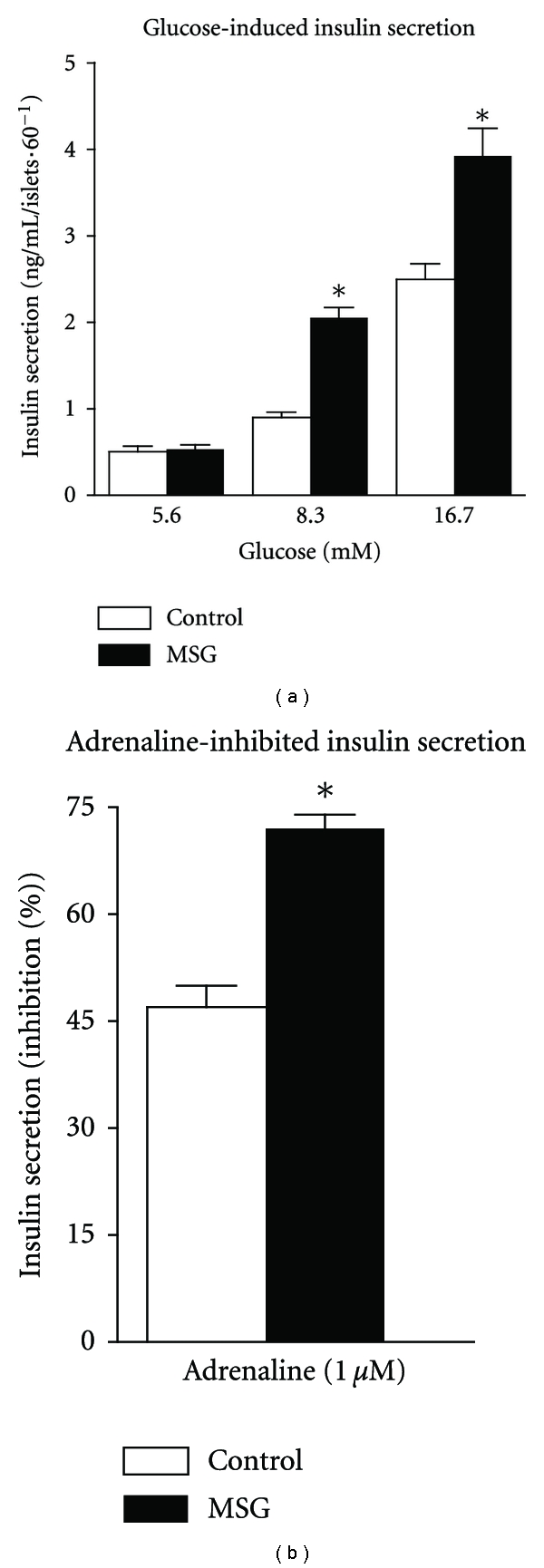
Glucose-stimulated insulin secretion and the inhibitory action of adrenaline on insulin secretion in MSG-obese rats. For each glucose concentration, 20 measurements, from islets isolated from 5 animals were performed. The data are shown as mean ± SEM. *P < 0.05 compared to control, by Student's *t*-test.

Figure 5 shows that isolated islets from both groups responded in a dose-dependent manner when stimulated by glucose; however, the dose-response curve was much steeper in islets from pre-diabetic obese animals than in lean animals. In low (5.6 mM) glucose, insulin secretion was similar between obese and control islets. On the other hand, pre-diabetic obese islets secreted 127 and 56% more insulin in high-glucose solutions of 8.3 and 16.7 mM, respectively, compared with control pancreatic islets (*P* < 0.05). Adrenaline inhibited glucose-stimulated insulin secretion in islets from both groups; however, this inhibition was more pronounced in islets obtained from pre-diabetic obese rats. The insulin secretion stimulated by 16.7 mM glucose was inhibited by 72 and 47% in pancreatic islets isolated from pre-diabetic obese and control animals, respectively, (*P* < 0.05).

**Figure 6:**
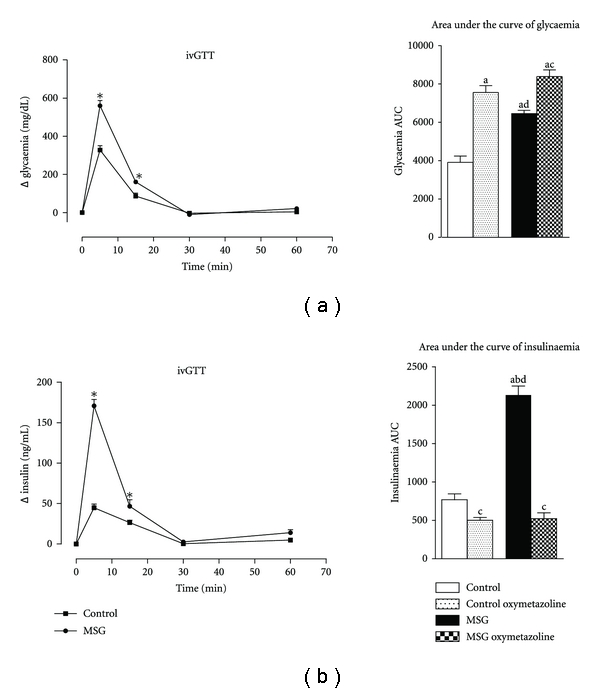
The effect of the *α*-adrenergic agonist oxymetazoline on glycaemia (a) and insulinaemia (b) during ivGTT in MSG-obese rats. Bars represent mean ± SEM (*n* = 8–10 animals per group). A Student's *t*-test or ANOVA was used where appropriate. *P < 0.05 compared to control. The letters over the bars represent a significant difference: a: *P* < 0.05 versus control; b: *P* < 0.05 versus control-oxymetazoline; c: *P* < 0.05 versus MSG; d: *P* < 0.05 versus MSG xymetazoline.

Figure 6 shows the results of the ivGTT test. Following a glucose load of 1 g/kg body weight, glycaemia increased in both groups; however, in pre-diabetic obese rats, the plasma glucose concentration was 78% higher than the control group (*P* < 0.05) at 5 min. The fasting glycaemia was restored at 30 min and was maintained until the latest time point measured (60 min) for both groups. The glucose intolerance in pre-diabetic obese rats can be clearly appreciated by considering the area under the curve (AUC) of glycaemia during ivGTT (Figure 6), which shows that obese animals had 65% higher glycaemia than control rats (*P* < 0.05). Moreover, oxymetazoline induced 30 and 93% more AUC glycaemia in pre-diabetic obese and control rats, respectively, (*P* < 0.05). 

Blood insulin levels during ivGTT are also presented in Figure 6 and show that plasma insulin levels increased in parallel with the rise in glucose levels in both groups; moreover, as with glucose levels, plasma insulin levels increased to a greater magnitude in obese rats. Plasma insulin levels were 280, 76, and 190% greater in pre-diabetic obese rats at 5, 15, and 60 min, respectively, relative to control rats (*P* < 0.05). Insulin AUC was 320% higher in obese rats compared with control rats (*P* < 0.05). The oxymetazoline effect on insulinaemia is also presented as AUC data in Figure 6. Oxymetazoline inhibited insulin levels by 76 and 35% in pre-diabetic obese and control animals, respectively, (*P* < 0.05).

## 4. Discussion

Energy homeostasis is regulated by the neural brain network, principally in the hypothalamus [6, 7]. In rodents, lesions in the ARC during the neonatal period lead to obesity in adulthood [33]. A frequently used model of obesity is neonatal administration of MSG; this treatment kills neurons in the ARC [33, 34]. These pre-diabetic obese rats have massive adipose tissue accumulation, delayed development, and elevated insulin levels [35–37], all of which were also found in the present study. Low levels of growth hormones are found in obese animals and are responsible for their reduced size and muscle mass. Insufficient circulating growth hormone levels also contributed to the reduced lipolytic capacity that obese animals exhibit [35, 38].

Reduced SNS activity plays an important role in several rodent models of obesity [15]. Genetic models of obesity exhibit decreased SNS activity to brown adipose tissue (BAT) and other peripheral organs [15, 39]. Levin demonstrated a reduced turnover of noradrenaline in organs, including the brain, of obesity-prone rats that preceded any increase in body weight [40]. SNS dysfunction has also been observed in obese humans. Saad et al. showed that sympathetic activity is an indicator of energy expenditure in Caucasians [41]. On the other hand, some authors have reported high SNS activation to the heart, blood vessels, and kidneys, which might be critical in the development of obesity-related hypertension [42]. Indeed, Davy and Orr reviewed SNS activity in human obesity and discussed the possibility of SNS outflow being differentiated according to its target organ, suggesting tissue specificity [10]. Direct evidence of weak SNS activity was demonstrated in the present study by recording the sympathetic firing rate in the superior cervical ganglion of pre-diabetic obese rats. However, data obtained from exercised, food-deprived, or fasted animals, as well as animals treated with a sympathoexcitatory agent, showed that when sufficiently stimulated, the SNS of MSG-obese rodents can activate sufficiently to drive physiological responses that are comparable to lean animals [21, 43–45]. 

Failed sympathoadrenal function has been described as an important feature in the onset of obesity [46–48]. According to Jocken and Blaak, impaired catecholamine-induced lipolysis can contribute to the adipose tissue development [49]. Data obtained in our laboratory have demonstrated that MSG-obese mice have decreased basal catecholamine secretion from the adrenal medulla compared to normal mice [50]. Indeed, MSG-obese mice have diminished adrenaline excretion and noradrenaline turnover in BAT compared to control mice [51, 52]. Nicotine significantly increases noradrenaline turnover, BAT oxygen consumption, and resting metabolic rate and significantly reduces body weight in MSG-obese mice without affecting food intake [43]. In support of a general reduction in SNS activity in obesity, we found decreased plasma circulating levels of adrenaline in pre-diabetic obese rats. Some authors have concluded that these results indicate low sympathetic activity that leads, at least in part, to decreased energy expenditure and fat mobilisation [51, 52]. 

Our results also show that obese rats have an increased catecholamine secretion response to all of the secretagogues that were tested in the adrenal medulla incubations. We believe that excitation-secretion coupling in adrenal chromaffin cells of pre-diabetic obese rats is physiologically preserved, as catecholamine secretion could still be induced by membrane depolarisation (triggered by elevated extracellular potassium), activation of cholinergic receptors (by carbachol), and calcium release from intracellular stores (triggered by caffeine). The relatively high total catecholamine content that we measured in obese rats may be the result of low SNS activity, which contributes to reduced catecholamine secretion.

TH catalyses the rate-limiting step in the biosynthesis of the catecholamines dopamine, noradrenaline, and adrenaline [53]. We previously reported that adult obese mice have decreased expression of the enzymes TH and dopamine beta-hydroxylase [25]. Similar TH results were observed in obese rats. The results of our current study also show that, despite this decrease in TH expression, catecholamine content is increased in the adrenal gland of prediabetic obese rats, which can be the result of decreased pre-synaptic stimulation of adrenal medulla chromaffin cells. Dysfunction of the SNS efferent signal produces low blood adrenaline levels, as observed in MSG-obese rats. Furthermore, TH expression may be considered a marker of cholinergic activity, as stimulation of acetylcholine receptors induces both TH enzyme activation and TH protein expression [54]. Acetylcholine released from the splanchnic nerve interacts with cholinergic receptors in the adrenal medulla, leading to activation of several protein kinases, including protein kinase C (PKC) [55], which catalyses TH phosphorylation and TH gene transcription [53]. 

No significant difference in *α*-PKC expression was observed in adrenal chromaffin cells of MSG-obese rats compared to controls; however, additional studies of PKC activity are needed to confirm this finding, as this kinase is activated primarily via the muscarinic signalling pathway. Indeed, Akaike et al. showed that PKC activation results in potassium channel phosphorylation, which reduces the channel's conductance and consequently induces membrane depolarisation, the latter of which contributes to increased catecholamine secretion [56]. 

Disruption of glycaemia homeostasis is a hallmark of obesity and is primarily attributed to insulin resistance [57–59]. We found that MSG-obese rats are glucose intolerant, confirming previously published results [36, 60, 61]. The elevated insulin levels found in MSG-obese rats can reflect insulin resistance. Molecular studies have demonstrated that MSG-obese animals have a decrease in signalling of their insulin-stimulated IRS/PI3K/Akt pathway in muscle and adipose tissue, which may be an important hallmark of insulin resistance of those animals [62]. Indeed, insulin binding to membrane receptors in liver, skeletal muscle, and adipocytes is decreased in MSG-obese rats [61]. 

The high insulin levels in pre-diabetic obese rats play a central role in fat cell proliferation and insulin resistance [62]. White adipose tissue in MSG-obese rats has elevated lipogenesis capacity and compensatory responsiveness to insulin by the Cbl and IRS2 pathways, which may mediate the insulin antilipolytic effect [62]. When MSG-obese rats undergo early vagotomy (at 30 days of life), hyperinsulinaemia is blocked, and fat accumulation is dramatically reduced [63]. Indeed, surgery to remove fat in 15-week-old MSG-obese rats improved their lipid profile and insulin resistance [64].

Furthermore, these results show that hyperinsulinaemia in obese rats is sustained by increased glucose responsiveness in isolated pancreatic islets. Increased responsiveness to glucose by pancreatic islets is also a feature of other obesity models and may be an adaptation to altered ANS tonus to endocrine pancreas with high parasympathetic activity [65, 66]. Early vagotomy prevents high-glucose-stimulated insulin secretion in MSG-obese rats [36]. Indeed, pancreatic islets from Zucker (*fa*/*fa*) rats, a genetic animal model of obesity, also have a high response to glucose, and islets from these animals have high levels of glucose utilisation and oxidation, which contribute to their observed hyperinsulinaemia [67]. 

Alpha-adrenergic activation has an inhibitory effect on glucose-stimulated insulin secretion, and this is accentuated in islets obtained from pre-diabetic obese rats, indicating that SNS tonus to islets may be reduced in obese rats. Islets isolated from both *ob*/*ob* mice and Zucker (*fa*/*fa*) rats, two genetic models of obesity, have higher responsiveness to the inhibitory action of noradrenaline [68, 69]. Cruciani-Guglielmacci et al. investigated an animal model of obesity obtained by a high-fat diet and reported that these animals developed severe glucose intolerance and insulin resistance that were due to increased glucose-induced insulin secretion. The authors further showed that injections of the *α*2-adrenergic receptor agonist oxymetazoline (even at low concentrations) reduced glucose-induced insulin secretion in the obese group; this effect was not observed in the control group. Low SNS activity in rats that received a high-fat diet may be responsible for their islet hypersensitivity to oxymetazoline, as concluded by the authors [70]. Our results also showed that oxymetazoline causes a profound decrease in blood insulin concentration in pre-diabetic obese rats, which suggests that insulin secretion is potently inhibited.

## 5. Conclusions

Our data support the hypothesis that MSG-obesity onset is dependent on reduced sympathetic tonus, including sympathoadrenal axis activity; however, physiologically, adrenal medulla function is preserved in these rats. Our data show that the low SNS activity observed in pre-diabetic obese rats leads to reduced basal catecholamine secretion from the adrenal medulla, which reflects a decreased concentration of plasma adrenaline. Indeed, the latter may contribute to the pancreatic islet's high sensitivity to adrenaline, indicating that the inhibitory effect of catecholamines on insulin secretion may be impaired in pre-diabetic obese rats. In addition to helping understand the mechanisms underlying obesity, this study may be helpful in developing strategies for treating and preventing the onset of obesity and diabetes by targeting sympathoadrenal axis activation.

##  Conflict of Interests

The authors have no real or perceived conflict of interests to disclose.

## Figures and Tables

**Table 1:** Effects of neonatal MSG treatment on adults rats.

	Control	MSG
Body weight (g)	278.60 ± 4.41	208.60 ± 3.31*
Lee index	281.20 ± 1.07	300.70 ± 1.62*
Epididymal fat pad (g/100 g bw)	1.58 ± 0.05	2.53 ± 0.06*
Retroperitoneal fat pad (g/100 g bw)	1.34 ± 0.04	2.10 ± 0.04*
Glycaemia (mg/dL)	101.80 ± 2.26	108.60 ± 6.99
Insulinaemia (ng/mL)	5.30 ± 0.72	15.06 ± 1.80*
Adrenal glands (mg)	23.9 ± 0.39	12.06 ± 0.36*
Adrenal glands (mg/100 g bw)	8.57 ± 0.12	5.79 ± 0.14*

Data represent mean **± **SEM. To all parameters, 20 rats were used for both groups. student's *t*-test was used. *P < 0.05 compared to control.
